# Extraction, GC-MS analysis, cytotoxic, anti-inflammatory and anticancer potential of *Cannabis sativa* female flower; *in vitro*, *in vivo* and *in silico*

**DOI:** 10.3389/fphar.2025.1546062

**Published:** 2025-02-11

**Authors:** Waqas Safir, Arif Malik, Haleema Saadia, Ayesha Zahid, Jinyao Li

**Affiliations:** ^1^ Xinjiang Key Laboratory of Biological Resources and Genetic Engineering, College of Life Sciences and Technology, Xinjiang University, Urumqi, Xinjiang, China; ^2^ School of Pain and Regenerative Medicine (SPRM), The University of Lahore, Lahore, Pakistan; ^3^ Faculty of Health Sciences, Equator University of Science and Technology, (EQUSaT), Masaka, Uganda; ^4^ Department of Biochemistry, Islam Medical College, Sialkot, Pakistan

**Keywords:** ADMET, cannabinoids, HeLa, humulene, molecular docking, PD-1/PD-L1, tetrahydrocannabinol

## Abstract

This work examines the anticancer activity, the anti-inflammatory nature, and the cytotoxicity of the ethanol extract obtained from the female flowers of *Cannabis sativa* L using molecular methods *in vitro*, animal testing *in vivo*, as well as computational methods and simulations *in silico*. From the GC-MS analysis, the following bioactive compounds were found: cannabidiol (CBD), tetrahydrocannabinol (THC), and humulene. The antiproliferative activities of the extract were determined on HeLa cells by using MTT, Crystal Violet, and Trypan Blue assays with an IC50 value suggesting 51%-77.6% lethality. The bioinformatics analysis of molecular docking proved significant ligand-protein interactions of CBD, THC, and humulene with cancer-associated proteins such as PD-1/PD-L1, TNF-α, and MMP-9. *In vivo*, breast cancer was first established in female Sprague-Dawley rats with 7,12-dimethylbenz(a)anthracene (DMBA) then treated with cannabinoids either singularly or in combination. Detailed treatment demonstrated that the use of the three cannabinoids simultaneously yielded the best anticancer and anti-inflammatory outcomes together with the best tumor reduction. The concentration of serum biomarkers of inflammation and tumor progression was substantially reduced in treated groups compared to the control group, which proves the synergistic effects of these cannabinoids in breast cancer therapy. This study emphasizes the importance of medical *Cannabis sativa* derivatives in cancer treatment.

## 1 Introduction

*Cannabis sativa* is the oldest cultivated plant and contains a lot of naturally useful components used by humans. It is used as a source of fuel, food, herbal body care products, and medicines to cure different diseases (Small and Marcus, 2002). It is a plant of the Cannabaceae family. It is an annual, dioeciously flower herb that belongs to central and eastern Asia. About 5,000 years ago, China started its cultivation to obtain fibre and oil from it (Happyana et al., 2013). Now, cannabis grows all over the world, including in Pakistan, America, Europe, and Brazil. Cannabis basically grows annually in warm and tropical areas of the world. Cannabis has three species: Cannabis indica, *Cannabis sativa*, and Cannabis ruderalis. *Cannabis sativa* gains high interest in the different fields of medicine as compared to other varieties (Guy et al., 2004). Cannabis has different types of chemicals known as cannabinoids (Alexander et al., 2009).

In women, cervical cancer appears as the second major issue and burden as a malignant disease. Cervical cancer is cancer of the cervix, as an abnormal division in the cervix increases the chances of spreading tumor cells to other parts without showing any symptoms. Cervical cancer grows and spreads slowly; patients can live for 5 years with this disease. At the last stage, symptoms such as bleeding from the vagina, vaginal pain, and pain during sexual activity appear. The human papillomavirus is the main cause of cervical cancer (Chang et al., 2021). HeLa is a cell line that has uncontrolled cell division, originating from cancer of the cervix. The Hela cell line is the first-ever cell line that shows continuity, as other cell lines could not persist or remain alive for more than a few weeks (Harro et al., 2001).

Breast cancer affects more than two million women annually and is the foremost cause of cancer death. Early diagnosis and treatment improve survival chances, but drug resistance remains a major challenge (Droste et al., 2023; Balkwill, 2006). Tumor cells shape the immune system, enhancing immunity to detection and eradication. In breast cancer immunotherapy, the PD-1-PD-L1 pathway, which inhibits T-cell function, is essential and is gaining interest (Bebars, 2023). T cells express the costimulatory protein PD-1, whereas cancer cells overexpress PD-L1, its ligand. When PD-1 and PD-L1 bind, both T-cell receptor expression and proliferation are blocked, which hinders the ability of the immune system to check on cancerous cells (Park and Lee, 2023). Cannabinoids, including cannabidiol (CBD) and tetrahydrocannabinol (THC), have renewed interest as novel pharmaceuticals and therapeutic approaches for treating cancer because of their anti-inflammatory, antitumorigenic, and immunomodulatory effects. CBD, a nonpsychoactive compound in cannabis, prevents the proliferation of cancer cells and regulates the immune system through interactions with different molecular signalling pathways. THC, the main psychoactive component of cannabis, also has anticancer properties and can induce growth, trigger apoptosis, and inhibit angiogenesis. Moreover, humulene, a sesquiterpene present in consumption and hops, has inflammatory and anticancer properties.

In breast cancer, the TME is inhibited by increased levels of cytokines, including TNF-α and IL-6, which are associated with both immunosuppression and tumorigenesis (Priya et al., 2025). Moreover, reactive oxygen species (ROS), which include 8-hydroxy-2′-deoxyguanosine (8-OHdG), advanced oxidation protein products (AOPPs), and advanced glycation end products (AGEs), have been implicated in cancer growth and recurrence as well as resistance to treatment. CBD, THC, humulene, and other cannabinoids and terpenes have shown potential for altering the TME by decreasing inflammatory responses, oxidative stress, and immunosuppressive signalling. These properties may also help ameliorate the effects of ROS and decrease 8-OHdG levels to restrain cancer development. Animal studies have shown that cannabinoids might act through the inhibition of the PD-1/PD-L1 pathway, which in this way reactivates T cells and stimulates immune-mediated cancer cell death (Bebars, 2023). The current aims to evaluate ligand-protein interaction by molecular docking, ADMET analysis, the anticancer, anti-inflammatory, and cytotoxic effects of the female flower Cannabis S. ethanol extract and its main active phytochemicals (THC, CBD, and Humulene).

## 2 Materials and methods

### 2.1 Sampling of cell line and plant extract

The HeLa cell line was acquired from The University of Lahore’s cell culture laboratory. These cell lines have been preserved in liquid nitrogen-filled cryo vials. Cryo vials were revived for further processing. For plant extract, the *Cannabis sativa* plant was collected from the field area of the University of Lahore. Plants were allowed to dry. Plant samples were grind into fine powder, and ethanol was added to the reagent bottle for up to 500 mL. After 1 week, the sample was filtered with Whatman filter paper. After filtration, the sample was dried via a rotary evaporater. The semi-dry sample was dried via a lyophilizer (freeze drying) machine.

### 2.2 GC-MS analysis

The GC–MS analysis of the plant extracts was carried out using the Agilent Technologies (7890 A) GC–MS triple quad system with EI and CI ion source as the instrument model. The apparatus features a non-polar column with an ID × 0.25 µm film and a 30 mm × 250 µm size, along with a DB 35 Ms capillary standard. Helium gas was used as a transporter gas and was adjusted to a column velocity flow of 1.0 mL/min for the 36-minute GC-MS experiment. While the injector operated at 250°C, the oven ran at 60°C for a holding time, then 10°C each minute to 310°C per 4 min. By comparing the compounds with real standards and their mass spectrum records from the National Institute of Standards and Technology (NIST 08. L) Library, their retention times were used to identify them.

### 2.3 Culturing of cell line

We thawed the cryo vials that were taken out of the liquid nitrogen cylinder. After that, DMEM-HG, 10% foetal bovine serum (FBS), streptomycin, and penicillin were added to the culturing flask containing the HeLa cell line. Sub-culturing of the cultivated HeLa cells was carried out whenever they reached 70%–80% confluence. Trypsin-EDTA was used to incubate the cells that were adhered to the culturing flask walls until they separated from the flask’s surface after they had been cleaned with 1X phosphate buffer saline. By looking at the flask under an inverted microscope, it was possible to ascertain that the cells had detached. The flask was filled with a few drops of FBS, which were thoroughly mixed by stirring. After centrifuging, the mixture was transferred to a 15 mL Falcon tube and centrifuged for 5 minutes at 2,000 rpm. Following centrifugation, the pellet was re-suspended and the supernatant was discarded.

### 2.4 Treatment of cell lines with ethanolic extract of *Cannabis sativa*

The HeLa cell line was cultivated on 6-well plates for Muse analysis and 96-well plates for IC50 calculation and cell viability. Two groups of the HeLa cell line were created. One group received treatment, whereas the other group received no treatment. The cultured cells were treated for 72 hours. Following a 72-hour period, the MTT assay, the trypan blue and crystal violet assay, and the Muse analysis were conducted using 96-well plates.

### 2.5 IC_50_ Calculation (MTT assay)

The cells were cultivated on 96-well plates and subjected to a 3-(4,5-dimethylthiazol-2-yl)-2,5-diphenyltetrazolium bromide (MTT) (Invitrogen Inc., United States) experiment in order to determine the IC50 of various *Cannabis sativa* extracts on the HeLa cell line. After washing the cell monolayer with phosphate buffer saline (PBS) (Invitrogen Inc., United States), it was incubated for 2 hours in 100 µL of complete media that contained 25 µL of MTT solution (Invitrogen Inc., United States). Living cells transformed tetrazolium into purple formazan, which was subsequently dissolved in dimethyl sulfoxide (DMSO) (Invitrogen Inc., United States). The absorbance of the solution was measured at 570 nm.

### 2.6 Cell viability (trypan blue) assay

Using trypan blue as a prohibiting agent for both living and dead cells, the IC50 value of each *Cannabis sativa* extract was utilised to evaluate cell viability. After three PBS washes, the treated and untreated HeLa cells were incubated for 15 min in trypan blue (Invitrogen Inc., United States). Following three PBS washes, the cells were examined under a microscope. Trypan blue-stained cells were regarded as dead.

### 2.7 Cell viability (crystal violet) assay

Cell viability was evaluated using the crystal violet staining method on the HeLa cell line, and the IC50 value of each *Cannabis sativa* extract was employed for this purpose. This procedure was carried out on a 96-well plate. The medium from each experimental group was removed from the plate’s wells and cleaned with PBS. Following washing, 2% ethanol and 0.1% crystal violet dye were applied to the wells until the entire surface was covered. For 15 minutes, it was incubated at room temperature. Wells were thoroughly rinsed to prevent cells from lifting off the well, and dye was disposed of with caution. After solubilising the stain with 100 µL of 1% SDS in each well, the wells were allowed for five to 10 minutes. Finally, absorbance on the microtiter plate was measured at 540 or 595 nm.

### 2.8 Count and viability kit

*Cannabis sativa* plant extract was added to HeLa cells grown in a 6-well plate, and the MTT test was used to determine the IC50 value. The automated cell analyser “Muse”TM (Merck-Millipore) was used to perform the count and viability kit (Cat. No. MCH100102). Following treatment, cells were centrifuged for 5 minutes at 2,000 rpm. After dissolving the pellet in the cell and discarding the supernatant, the viability reagent was left to wait for 5 minutes. The “Muse”TM automated cell counter and analyser was used to count the cells.

### 2.9 Annexin V and Dead Cell Kit

*Cannabis sativa* plant extracts were added to HeLa cells grown in 6-well plates, and the MTT test was used to determine the IC50 value. The automated cell analyser “Muse”TM (Merck-Millipore) was used to perform the Annexin V and Dead Cell Kit (Cat Number: MCH100105). Following treatment, cells were centrifuged for 5 minutes at 2,000 rpm. The pallet was dissolved in Annexin V, the supernatant was disposed of, and the apoptosis reagent was left to dissolve for 5 minutes. The “Muse”TM automated cell counter and analyser was used to count the cells.

### 2.10 Animals

One hundred mature female Sprague–Dawley rats weighing 180–220 g at 8 weeks of age were used in this study. The rats were kept under conventional circumstances, which included a 12-hour artificial light‒dark cycle at a temperature of 22°C ± 2°C and humidity. Animals were provided with a nutritionally balanced diet and fresh water *ad libitum*, and all experimental compounds or chemicals were administered in carefully calculated doses as per the guidelines approved by the Institute of Molecular Biology and Biotechnology Ethical Review Committee. The rats were assigned to ten groups in equal proportions in accordance with the experimental design described below.

#### 2.10.1 Tumor induction

Breast cancer was initiated in the rat by a single administration of 7,12-dimethylbenz(a)anthracene (DMBA) at a dose of 80 mg/kg of body weight in soy oil through intragastric gavage (Karnam et al., 2017; Adelegan et al., 2023; Akhouri et al., 2020). Groups B–J received DMBA treatment for breast tumors, whereas Group A served as the untreated control (Table 1).

**TABLE 1:** Experimental design.

Groups	Treatments
A	Control
B	DMBA
C	DMBA + Cannabidiol (CBD)
D	DMBA + Tetrahydrocannabinol (THC)
E	DMBA + Humulene (HL)
F	DMBA + Cannabidiol + Tetrahydrocannabinol
G	DMBA + Cannabidiol + Humulene
H	DMBA + Tetrahydrocannabinol + Humulene
I	DMBA + Cannabidiol + Tetrahydrocannabinol + Humulene
J	DMBA + Paclitaxel

• A single dose of 80 mg/kg of 7,12-dimethylbenz(a) anthracene (DMBA) diluted in soy oil (1 mL) given intragastrically by gavage.

• Cannabidiol, tetrahydrocannabinol, and humulene (100 mg/kg B. Wt./day) for 4 months.

• Paclitaxel^©^10 mg/kg B. Wt once a week for 4 weeks.

#### 2.10.2 Treatment administration

For 4 months after the administration of DMBA, CBD, THC, and HL were given orally at a dosageof 100 mg/kg body weight per day. Paclitaxel, a standard chemotherapeutic medication, was administered to Group J once a week for 4 weeks at a dose of 10 mg/kg body weight (Petersen et al., 2024). All the rats were sacrificed after 4 months of treatment, and samples of their blood and tumors were taken for molecular and biochemical analysis.

#### 2.10.3 Histopathology

The animals were euthanized in a CO2 chamber, and gross *postmortem* examinations were performed. The weights of the unresectable breast tumors were measured. The findings were recorded. After being removed, a portion of each tumor was preserved in 10% formalin and prepared for paraffin embedding. Hematoxylin‒eosin (H&E) was used to stain the 5 mm sections, which were then observed under a light microscope.

#### 2.10.4 Biochemical assays

Enzyme-linked immunosorbent assays and immunohistochemical analysis were used to determine the concentrations of PD-1 and PD-L1, the main variables in tumor tissues. Commercially available ELISA kits (Abcam) were used to quantify the levels of 8-OHdG (8-hydroxy-2′-deoxyguanosine), TNF-α (tumor necrosis factor-alpha), MMP-9 (matrix metalloproteinase-9), and IL-6 (interleukin-6). Advanced oxidation protein products (AOPPs) and advanced glycation end products (AGSs) were estimated via spectrophotometric techniques.

#### 2.10.5 Ethics approval

It has been confirmed that the experimental data collection conforms to relevant institutional, national, and international guidelines and legislation with the appropriate approvals from the Institute of Molecular Biology and Biotechnology’s (IMBB) Ethical Review Committee, The University of Lahore, Pakistan.

### 2.11 *In silico* study

#### 2.11.1 Data collection

The active phytocompounds Cannabidiol (CBD), tetrahydrocannabinol (THC), Humulene and the standard drug paclitaxel were downloaded from the PubChem database in SDF format, whereas the receptors PD1 (4ZQK), PD-L1 (5NIU), MMP-9 (1GKC), IL-6 (1ALU), TNF-α(1TNF), AGEs (2MOV), and AKT1 (3O96) were downloaded in PDB format (www.pdb.org/pdb).

#### 2.11.2 Protein preparation

Receptor proteins were opened in Discovery Studio software 2021 (https://discover.3ds.com/discovery-studio-visualizer-download) to eliminate unwanted materials to prepare the protein for molecular docking. The protein was stripped of all heat atoms, water molecules, and bound ligands; hydrogen atoms were then added and stored in PDB format (Bodily et al., 2011).

#### 2.11.3 Ligand preparation

After being obtained from the PubChem database, ligands in SDF format were uploaded into PyRx’s Open Babel window and transformed into pdbqt format (Qadir et al., 2025; Zahid et al., 2023).

#### 2.11.4 Molecular docking

The PyRx bioinformatics tool (https://pyrx.Sourceforge.io/) was used (Quazi et al., 2022) to examine the ligand–protein interactions of three chosen candidate compounds and a standard paclitaxel against PD-1, PD-L1, MMP-9, IL-6, TNF-a, AGEs, and AKT1. The receptor proteins PD1, PD-L1, MMP-9, IL-6, TNF-a, AGEs, and AKT1 were each uploaded into PyRx separately and transformed into macromolecules. The Open Babel window was used to upload each ligand molecule, and the energy was reduced and transformed into pdbqt format. After Vina Wizard was used to create a grad box (https://vina.scripps.edu/), docking was performed (Zahid et al., 2023). The dimensions of the grad box for PD1 X:38.20Y:37.73Z:28.19, PD-L1 X:46.02Y:86.89Z:53.20, MMP-9 X:53.88Y:46.73Z:75.43, IL-6 X:47.56Y:50.08Z:40.07, TNF-a X:63.83Y:67.18Z:64.15, AGEs X:28.98Y:51.61Z:33.20and AKT1 X:54.11Y:66.53Z:66.78 followed. The data were visualized via Discovery Studio Version 4.5 (https://www.discngine.com/disco very-studio), which included 2D structure, hydrogen bonding, and other types of bonding interactions.

#### 2.11.5 Drug-likeness and ADME

The SwissADME online server (http://www.swissadme.ch/) was utilized to verify ADME analysis and drug-likeness. Absorption, distribution, metabolism, and excretion are all included in ADME. To examine drug-likeness and ADME characteristics, canonical SMILES of ligand compounds were obtained from PubChem and put into SwissADME.

#### 2.11.6 Toxicity

To determine toxicity, canonical SMILES of each ligand were obtained from the PubChem database and placed into admetSAR (http://lmmd.ecust.edu.cn/admet sar2).

### 2.12 Statistical analysis

One-way analysis of variance was used to analyze the data, after which a Tukey test was used for *post hoc* comparisons. The level of statistical significance was set at p < 0.05. The data were analyzed and are presented as the means ± standard deviations (SDs).

## 3 Results

### 3.1 GC-MS analysis

**FIGURE 1:**
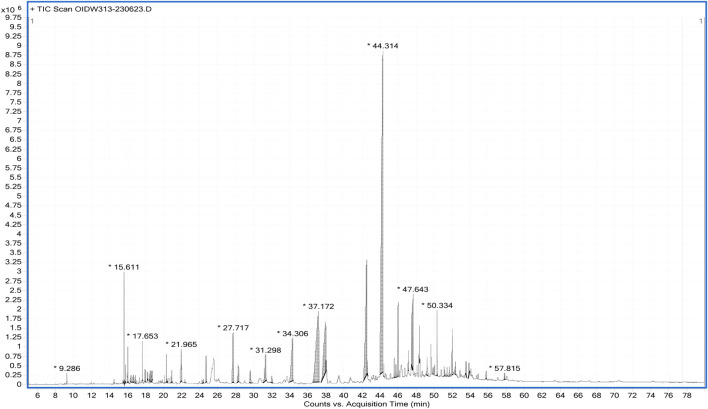
GC-MS Chromatogram of *Cannabis S.* Female flower ethanol extract.

The spectra from GC-MS analyses and those from the NIST reference library were compared. The phytocompounds were identified using molecular formula (MF), peak value, retention time (RT), and chemical names. It is possible to compare the chemical patterns in the generated mass spectra with reference spectra from the data repository. The ethanol extract’s GC-MS spectrum analysis revealed the presence of phytocompounds that significantly enhance *Cannabis sativa’s* therapeutic qualities. A total of fifty two compounds were detected by GC-MS analysis, and their chromatogram is given below (Figure 1). The main active phytochemicals that are reported for their medicinal uses in different research are THC, CBD, humulene, stigmasterol, *etc.*, which are our main focus of this study, and were detected by GC-MS of ethanol extract.

### 3.2 MTT assay of ethanolic extract of *Cannabis sativa*

**FIGURE 2:**
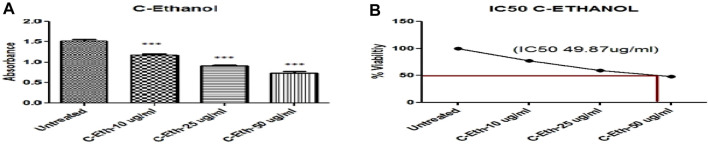
**(A)** Ethanolic Plant Extract (C-Eth) Displayed Anticancer Activity on Cervical Cancer Cells (HeLa Cell) (***) Showed Significance. **(B)** The percentage of viability Illustration of the Evaluation of the IC50 Value of *Cannabis sativa* Ethanolic Extract. The absorbance of the solution was measured at 570 nm after it was incubated for 2 hours in 100 µL of complete media with 25 µL of MTT solution (Invitrogen Inc., United States).

HeLa cells were treated with an ethanolic extract of *Cannabis sativa* at three different doses: 10, 25, and 50 μg/mL. For the sake of complete and competent results, the cells were analyzed by applying MTT reagent. When results were plotted against the controlled or untreated group of cells, it was observed that there was a significantly higher apoptosis rate, as shown in Table 2 and Figure 2A.

**TABLE 2:** Cytotoxicity of ethanolic extract of *Cannabis sativa*.

Cell lines	Apoptosis rate
Untreated (±SEM)	1.55 ± 0.0284
C-Eth (±SEM) 10 ug/mL	1.18 ± 0.0292
C-Eth (±SEM) 25 ug/mL	0.907 ± 0.0513
C-Eth (±SEM) 50 ug/mL	0.733 ± 0.0663

### 3.3 IC_50_ assessment via MTT assay of ethanolic extract of *Cannabis sativa*

The IC_50_ was observed through the MTT assay, which is a reliable method for cell cytotoxicity. Figure 2B shows the plot of the percentage (%) cytotoxicity of the ethanolic extract of the *Cannabis sativa* plant treated HeLa cells in a dose-dependent mode.

### 3.4 Cell viability assay (trypan blue, crystal violet)

**FIGURE 3:**
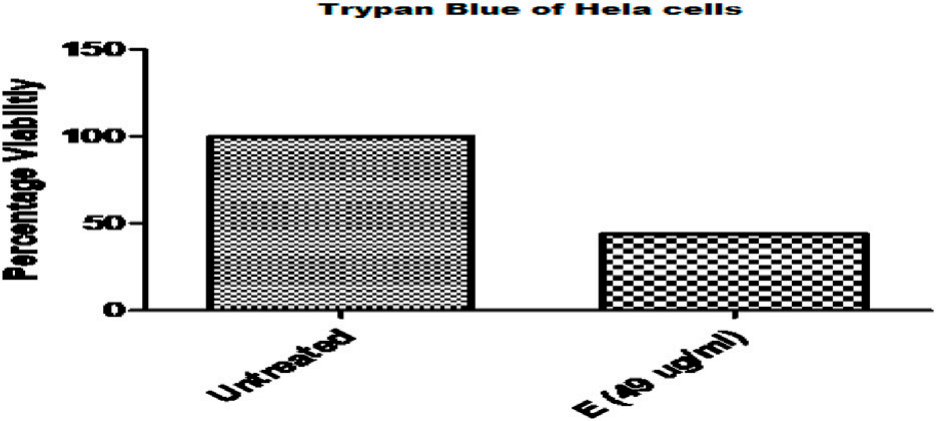
Comparative Analysis of Ethanolic Plant Extract on Cervical Cancer Cells (HeLa cell) By Trypan Blue Assay.

Trypan blue staining was used to administer ethanol plant extract to HeLa cancer cells. Compared to untreated HeLa cells, a notably greater number of blue-colored cells were seen in the treated group, suggesting that there were more dead cells (Figure 3).

**FIGURE 4:**
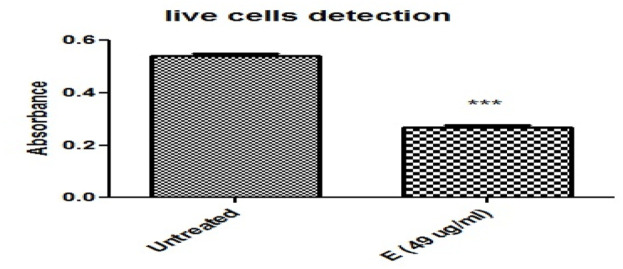
Ethanolic plant extract’s effects on HeLa cervical cancer cells are compared using the Crystal Violet Assay (*, ***) demonstrated the significant differences between the treated and untreated groups. Cell viability was evaluated using the crystal violet staining method on HeLa cell lines, and the IC50 value of each *Cannabis sativa* extract was employed for this purpose. Absorbance was measured on a microtiter plate at 540 and 595 nm.

The HeLa cell line’s crystal violet staining was also used to measure cell viability. Compared to untreated HeLa cells, cancer cells treated with ethanolic plant extract displayed fewer live cells (Figure 4; Table 3).

**TABLE 3:** Illustrates the comparison of untreated and treated groups. Measure of precision for an estimated population mean. *SEM is* the standard deviation of the sampling distribution, including treated and untreated samples.

HeLa groups	(±Sem)
Untreated	0.540 ± 0.01732
Ethanolic extract	0.2680 ± 0.0130

### 3.5 Muse analysis

**FIGURE 5:**
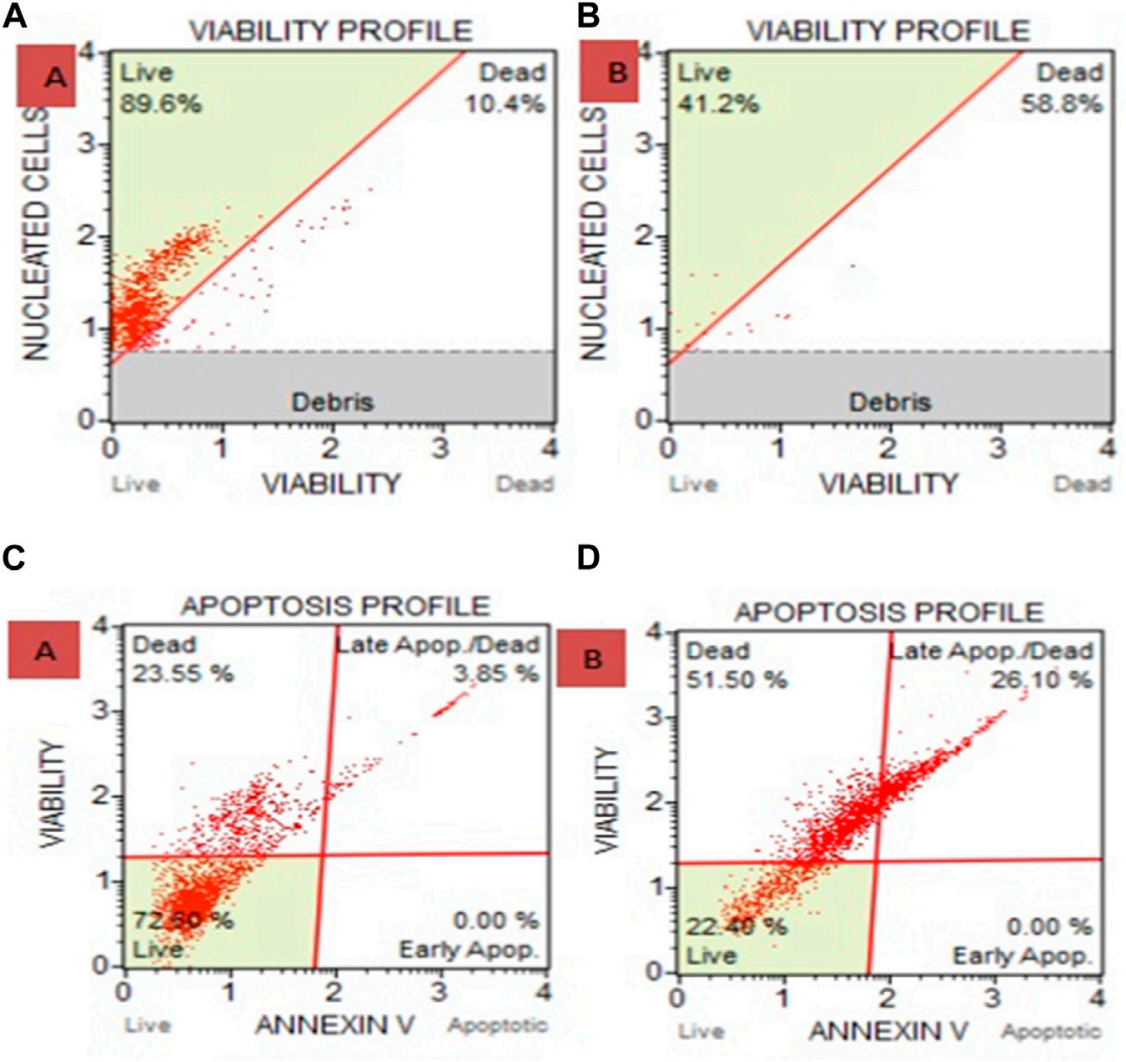
**(A)** Cell and Count Viability Assay where **(A)** is Untreated and B is Ethanolic Extract. 41.2% of the cells in the ethanolic extract treatment group were viable, compared to 89.6% in the untreated group. **(B)** after treating with ethanolic extracts show 58.8% death of carcinoma cells. **(C)** Apoptosis for HeLa cells treated with ethanolic extract of *Cannabis sativa* where **(C)** is untreated and **(D)** is ethanolic extract treated shows 51.5% death and via late apoptosis it shows 26.10% carcinoma cell death. Overall apoptosis initiate 77.6% death in carcinoma.

Figure 5A displays the cell count as viability findings after cells were treated with a plant ethanolic extract. The results indicate cell death. In comparison to the untreated group, HeLa cells treated with an ethanolic extract of *Cannabis sativa* killed more cells, and few living cells were seen. 89.6% of the cells in the untreated group were viable, while 41.2% of the cells in the ethanolic extract treatment group were viable.

### 3.6 Apoptosis through muse

HeLa cells treated with ethanolic extract showed 77.6% dead cells as compared with the untreated group, where 22.40% were live cells (Figure 5B).

### 3.7 Biochemical analysis

**FIGURE 6:**
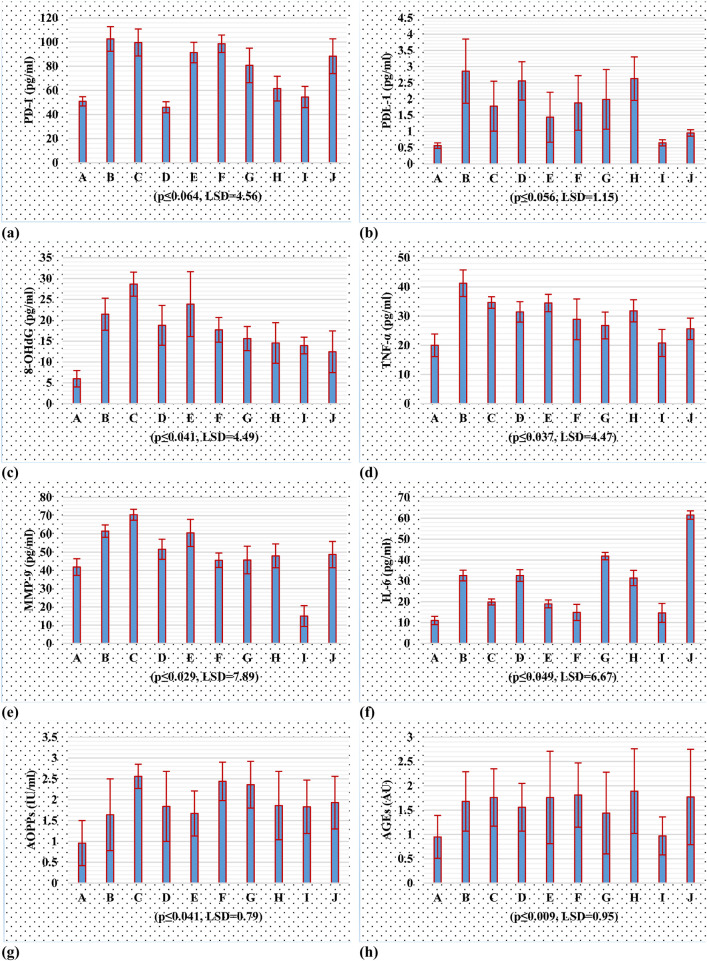
Tumor inhibitory potential of cannabinoids, in PD-1/PD-L1 and oxidative stresss; **(A)** PD-1 **(B)** PD-L1 **(C)** 8-OHdG **(D)** TNF-α **(E)** MMP-9 **(F)** IL-6 **(G)** AOPPs **(H)** AGEs, using rat breast cancer model.

The study examined (Figure 6; Supplementary Table S1) the regulation of PD-1, PD-L1, MMP-9, AGEs, AOPPs, IL-6, 8-OHdG and TNF-α expression in a rat model of breast cancer induced by DMBA. The results showed that CBD, THC, and HL significantly reduced PD-1 expression levels compared to the control group. THC treatment decreased PD-1 levels (45.99 ± 4.57), suggesting it may reverse tumor immune escape. HL treatment showed a modest reduction, but not statistically significant (91.33 ± 8.50). The combination of CBD and THC resulted in a similar PD-1 level, but not statistically significant (98.64 ± 7.22). The expression of PD-1 in the combined group was significantly downregulated (54.49 ± 8.78), suggesting synergistic inhibition of PD-1. The paclitaxel group had a lower PD-1 level (88.29 ± 14.39) than DMBA but still significantly higher than the THC and both combination treatment groups. Results showed that the DMBA-induced breast cancer group showed a significant increase in PD-L1 expression (2.86 ± 0.99), confirming tumor resistance mechanisms. CBD treatment reduced PD-L1 expression to 1.78 ± 0.77, while THC treatment resulted in a higher expression level (2.56 ± 0.59) but lower than the DMBA group. HL group showed elevated PD-L1 expression, suggesting it may promote tumor resistance. The combination of CBD, THC, and HL significantly reduced PD-L1 levels (0.651 ± 0.095), indicating a promising inhibitory effect. The results of 8-OHDG showed that CBD, THC, and HL significantly reduced oxidative stress in DMBA-induced breast cancer rats. The greatest decrease was achieved when all three compounds were used together (13.94 ± 1.99), suggesting that these active substances could be used as a basis for designing adjuvant therapies to overcome tumor resistance mechanisms by modifying redox processes. However, more extensive studies are needed to understand the mechanism by which RSV influences these effects and their significance in clinical management of breast cancer. The results showed that CBD, THC, and HL significantly decreased TNF-α concentration in the blood of the DMBA-induced breast cancer model rats, but the changes were not significant. When all three cannabinoids were used simultaneously, the greatest decrease in TNF-α level was detected (20.80 ± 4.64), suggesting that their effects may be synergistic when used to control inflammation related to cancer formation. The TNF-α level in the paclitaxel-treated group was also reduced (25.62 ± 3.67), although it was still elevated compared to the control group. The control group showed normal MMP-9 levels, while the cancer group showed a significant increase in MMP-9 levels (61.55 ± 3.40). CBD treatment led to a notable increase in MMP-9 levels (70.50 ± 2.98), suggesting that CBD may exacerbate MMP-9 activity. THC treatment resulted in lower MMP-9 levels (51.59 ± 5.50) but still elevated levels. The combination of CBD, HL and THC led to a significant reduction (14.99 ± 5.69) in MMP-9 levels, suggesting a potential synergistic effect. Paclitaxel treatment resulted in lower MMP-9 levels (48.68 ± 7.21) but still greater than the control group. The results showed that DMBA-induced BC group had higher IL-6 concentrations, indicating protumor inflammation. CBD treatment led to lower IL-6 levels (19.87 ± 1.44) but remained elevated. THC treatment resulted in similar levels (32.56 ± 2.80) but with sustained inflammation. HL treatment had lower levels (18.95 ± 1.89) but still higher than the control group. Combination treatment with CBD and THC resulted in an inhibitory effect, but not statistically significant (14.85 ± 3.87). The study suggests that DMBA-induced breast cancer model rats respond differently to treatment with CBD, THC, or HL in terms of IL-6 levels. The results showed that CBD, THC, and HL have different effects on AOPP levels in a DMBA-induced breast cancer rat model. The control group had normal oxidative stress levels, while the DMBA-induced breast cancer group showed increased AOPP levels. CBD treatment increased AOPP levels (2.56 ± 0.29), suggesting that CBD alone might increase oxidative protein damage. THC (1.84 ± 0.84) and HL (1.67 ± 0.54) treatments maintained AOPP levels, while their combination with CBD or HL did not significantly affect these changes. The results showed that the DMBA-induced breast cancer group had significantly higher AGE levels (1.68 ± 0.61) than the control group, indicating increased oxidative stress and inflammation. CBD treatment resulted in higher AGE levels (1.76 ± 0.59) than the control group, while THC treatment showed slightly lower levels (1.56 ± 0.49). Humulene treatment (1.76 ± 0.95) showed similar levels to CBD. Combination therapy with all three compounds led to even lower levels (0.97 ± 0.39), suggesting a possible protective effect. The paclitaxel treatment group showed no significant reduction in AGEs (1.77 ± 0.98). The results suggest that cannabidiol, tetrahydrocannabinol, and humulene, when used in combination, have a significant effect on AGE levels in the tissues of rats with DMBA-induced breast cancer.

### 3.8 Histopathology

**FIGURE 7:**
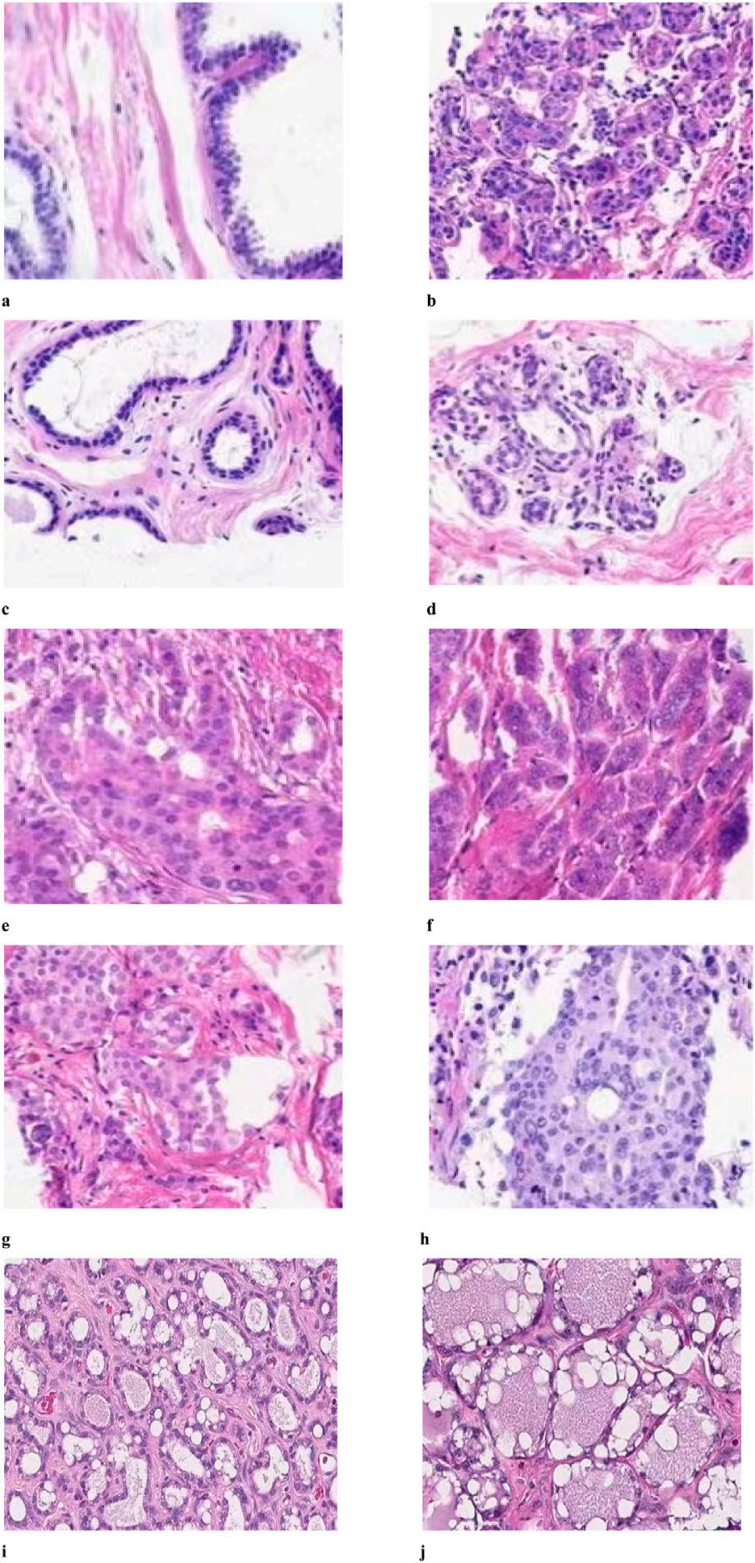
Histopathology of mammary tissue from rats in groups **(A–J)**.

This study examined the impact of herbal treatment on breast cancer tissue in a rat model. The tissues were stained with H&E, which revealed different responses. Figure 7. A served as a control and was not treated with DMBA. Figure 7B shows the tumor-induced tissue that was not treated with any type of therapy. Figure 7C reflects a moderate response, where the treatment shows some success in containing tumor growth but not to the extent observed in Image A. The tissue in Figure 7D shows a grouping of cells with visible nuclei, suggesting a dense, potentially malignant region. In studies targeting PD-1/PD-L1 pathways, such clusters might indicate the presence of tumor cells attempting to evade immune detection by expressing PD-L1, which suppresses immune responses. Herbal treatment might aim to inhibit PD-L1 expression, thereby reactivating immune cells to target and kill these tumor cells. In Figure 7D The disorganized, densely packed structure of the cells suggests a high-grade tumor area where the tissue structure is heavily disrupted. The cells are likely to proliferate rapidly, and the lack of organized structure implies aggressive tumor behavior. Figure 7E compared to 3D, the cells in this image are more uniformly spaced and slightly less crowded. This could indicate a transition zone where some tumor cells respond to the treatment, resulting in less aggressive growth. Herbal treatment may work by reducing oxidative stress, slowing the rate of proliferation, and potentially enhancing immune cell activity. Figure 7F depicts a fibrotic or stromal region that might show structural changes due to the antioxidative and immune-enhancing effects of the herbal treatment. Figure 7G reflects a potential intermediate treatment response and shows intermediate organization with visible stromal components, which might indicate reduced tumor aggression. This finding suggests that treatment may reduce oxidative stress or impact the tumor microenvironment, inhibiting rapid cell growth. Figure 7H shows moderate efficacy, with some signs of treatment impact, and displays characteristics that suggest that the treatment is starting to impact the tumor structure, although some regions may still be proliferating. Figure 7I suggest a strong treatment effect, with substantial cell death and vacuolation, indicating successful inhibition of tumor cell survival mechanisms.

### 3.9 Tumor weight

**FIGURE 8:**
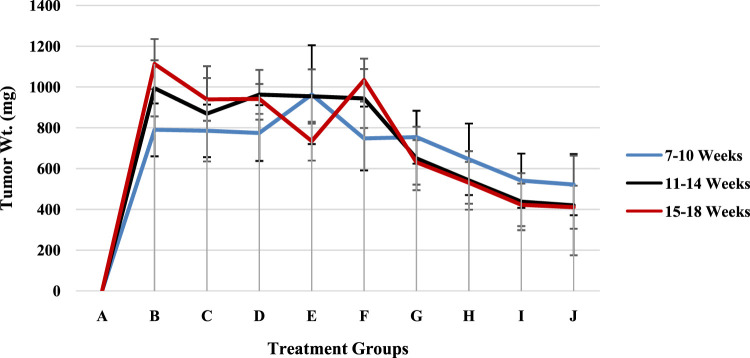
The tumor weights of the rat models in groups A–J.

The effects of treatment duration on groups A through J over a few months are summarized and are shown (Figure 8; Supplementary Table S2) as the mean values with standard deviations (Mean ± SD). Three treatment durations—7–10, 11–14, and 15–18 weeks—are highlighted in Figure 8. The least significant difference (LSD) criterion for group comparisons is 21.58, and for time period comparisons, it is 102.09. A threshold of 0.017 for the p value was used to determine statistical significance. With increases from week 7–10 to week 11–14 and some fluctuations in the 15–18 week period, groups B through F had higher mean values overall. Overall, Groups G through J presented lower mean values, with declining trends over the course of the weeks, particularly for Groups H, I, and J.

### 3.10 ADME analysis

An ADME study was carried out utilizing Swiss ADMET to ascertain a number of attributes, including the physical features, lipophilicity, Lipinski, and solubility of bioactive components. Given how frequently medication candidates fail clinical trials, ADMET plays a crucial role in drug development. For candidates for oral drugs, no more than two infractions are permitted. The Lipinski rule of five (≤10 H-bond acceptors, Mol. weight ≤500 Da, <5 logP, and molar refractivity 40–130) was used to assess whether specific compounds may be used as drugs. According to the data in Table 4, all of the chosen substances passed the Lipinski rule of five, with the exception of CBD, THC, and humulene, which had only one infraction. The common medication paclitaxel, however, has two effects. In terms of gastrointestinal absorption, humulene and paclitaxel have low GI values, but two compounds—CBD and THC—have high GIs. The log values for water solubility fall between −10 and 0, indicating distinct solubility categories: −10, −6, −4, −2, and 0 for insoluble, slightly soluble, soluble, very soluble, and very soluble, respectively, to be exact (Abdellatif et al., 2021). Table 4 shows that every compound in the chosen candidates is within the range of solubility.

**TABLE 4:** ADME analysis and drug-likeness results of selected and standard drug compounds.

Properties	THC	Cannabidiol	Humulene	Paclitaxel
MW	314.46	314.46	204.35	853.91
Heavy atoms	23	23	15	62
Aromatic heavy atoms	6	6	0	18
Rotatable bonds	4	6	0	15
H-bond acceptors	2	2	0	14
H-bond donors	1	2	0	4
MR	97.91	99.85	70.42	218.96
TPSA	29.46	40.46	0	221.29
Silicos-IT Log P	5.41	5.42	3.91	4.59
GI absorption	High	High	LOW	LOW
Silicos-IT LogSw	−5.93	−5.41	−3.52	−8.8
Lipinski violations	1	1	1	2

### 3.11 Toxicity

All the components that might act as medication candidates were examined via the online Protox-II server to determine their toxicity, including their cytotoxicity, carcinogenicity, and hepatotoxicity. To ascertain the adverse effects of a compound on people, plants, and the environment, toxicity prediction is crucial in the medication design process. Traditional methods involve the use of several animal studies to determine the toxicity of the substance, which is expensive, time-consuming, and fraught with ethical issues. Comparatively speaking, computer-aided toxicity prediction is less expensive and time-consuming, yields results more frequently, and requires fewer experimental biological studies. All of the chosen candidates—cannabidiol (CBD), tetrahydrocannabinol (THC), and humulene—are nontoxic and have the potential to be used as drugs, according to Table 5.

**TABLE 5:** Toxicity results of selected and standard drug compounds.

Molecules	Hepatotoxicity	Carcinogenicity	Cytotoxicity
Humulene	Inactive	Inactive	Inactive
THC	Inactive	Inactive	Inactive
CBD	Inactive	Inactive	Inactive
Paclitaxel	Inactive	Inactive	Active

### 3.12 Molecular docking

**FIGURE 9:**
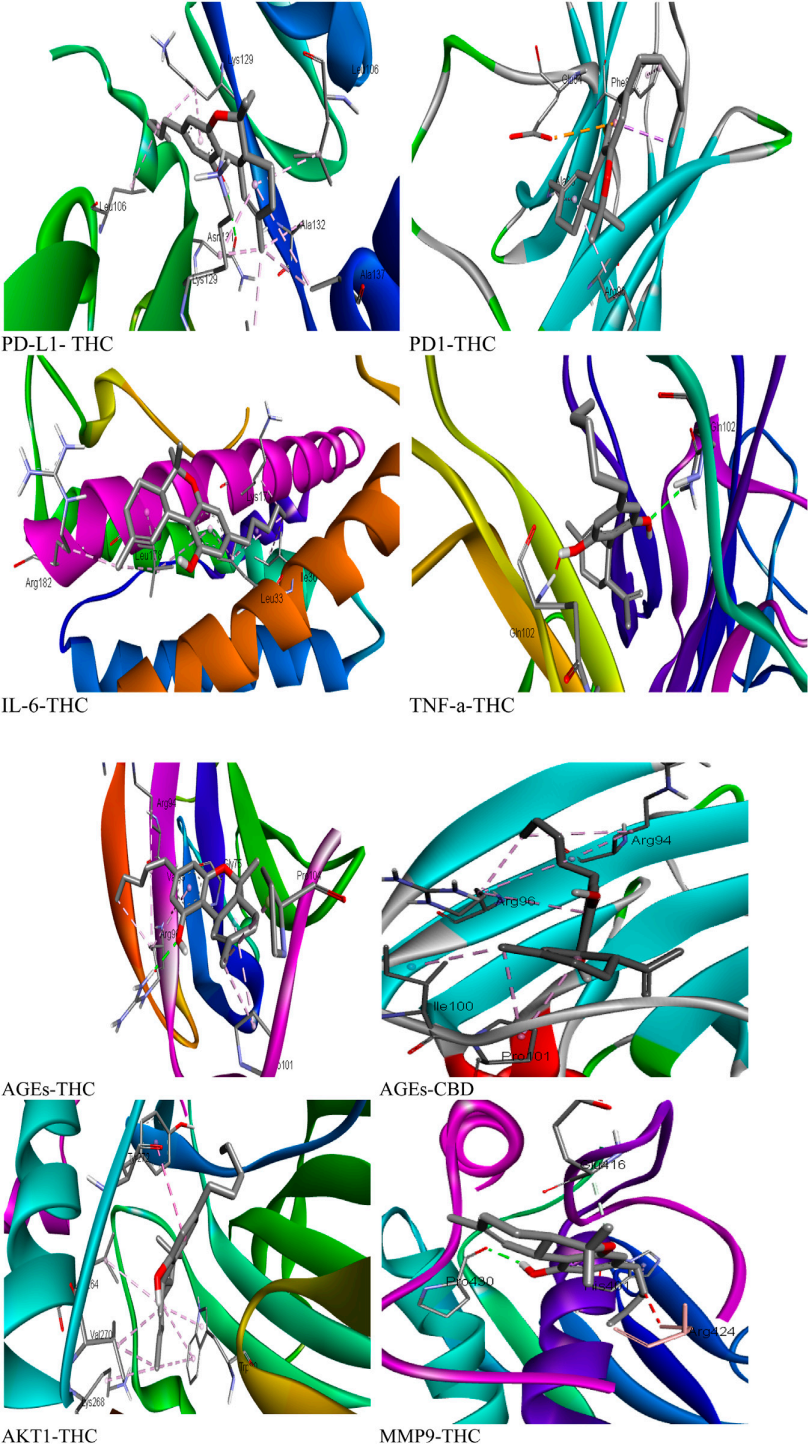
3D interactions of phytocompounds with the highest binding affinity for target proteins.

To assess the therapeutic potential of a few chosen compounds as antioxidative stress agents and anticancer medicines, molecular docking was performed against PD1, PD-L1, MMP-9, IL-6, TNF-α, AGEs, and AKT1 (Figure 9). Molecular docking is widely employed in contemporary drug discovery and plays a crucial role in ligand–protein and protein–protein interactions to determine the optimal position and binding sites of receptors to which a ligand molecule interacts. Table 6 shows that all 3 drug candidates have potential for binding with specific receptors, but tetrahydrocannabinol (THC) and cannabidiol (CBD) have promising binding affinities for each target, which is almost equal to or greater than the binding score of paclitaxel. These compounds interact with each target through hydrogen and hydrophobic interactions. The amino acid residue interaction analysis revealed (Table 7) that ASN131, ALA137, VAL29, LYS129, ALA132, and LEU106 of PD-L1; ASP117, ARG69, PHE95, and PRO72 of PD1; PRO430 and GLU416; HIS401 of MMP-9; ARG182, LEU178, LEU33, ILE36, and LYS171 of IL-6; GLN102 of TNF-a; ARG69, GLY75, ARG94, PRO101, PRO104, ARG96, and ILE100 of AGEs; and TYR272, LYS268, VAL270, LEU264, and TRP80 of AKT1 interact with tetrahydrocannabinol and cannabidiol.

**TABLE 6:** Molecular docking of selected phytocompounds and standard drugs.

Target	THC	CBD	Humulene	Paclitaxel
PD1	−6.0	−5.3	−5.2	−6.2
PD-L1	−8.2	−7.6	−5.6	−8.4
AKT1	−9.6	−8.3	−7.1	−8.5
TNF-a	−7.6	−7.7	−6.8	−7.9
MMP-9	−8.2	−7.7	−5.6	−8.2
IL-6	−6.8	−6.0	−6.2	−6.8
AGEs	−6.9	−6.4	−5.6	−5.9

**TABLE 7:** Type of bonding and amino acid residues of target proteins that interact with drug compounds.

S.No	Receptors	Compounds	Binding affinity	Bonding type	Bond category	Amino acid residues
1	PD-L1	THC	−8.2	Hydrogen	Conventional	ASN131
				Hydrophobic	Alkyl	ALA137, VAL29,LYS129, ALA132, LEU106
					Pi-Alkyl	LYS129
2	PD1	THC	−6	Hydrogen	Conventional	ASP117
				Hydrophobic	Pi-Sigma	ARG69
					Pi-Alkyl	PHE95
					Alkyl	PRO72
3	MMP9	THC	−8.2	Hydrogen	Conventional	PRO430
					Carbon	GLU416
				Hydrophobic	Pi-Alkyl	HIS401
4	IL-6	THC	−6.8	Hydrophobic	Alkyl	ARG182, LEU178, LEU33, ILE36, LYS171
					Pi-Alkyl	LEU33, LEU178
5	TNF-a	CBD	−7.7	Hydrogen	Conventional	GLN102
6	AGEs	THC	−6.9	Hydrogen	Conventional	ARG69
					Carbon	GLY75
				Hydrophobic	Amide-Pi Stacked	VAL95
					Alkyl	ARG94, PRO101, PRO104, ARG96
					Pi-Alkyl	ARG96
		CBD	−6.4	Hydrophobic	Alkyl	ILE100, PRO101, ARG94, ARG96
					Pi-Alkyl	ARG96
7	AKT1	THC	−9.6	Hydrophobic	Pi-Pi Stacked	TYR272
					Alkyl	LYS268, VAL270, LEU264
					Pi-Alkyl	TRP80

## 4 Discussion

The current study confirmed the presence of THC, CBD, and humulene in female *Cannabis S.* flower ethanol extract by GC-MS analysis. They are considered to be the most active phytochemicals which are reported for leaves extract of the said plant in different studies. In this study, notable effects of cannabinoids were identified in upregulation of apoptosis, autophagy, and downregulation of proliferation and invasion in carcinoma cells of cervix cancer on HeLa. Statistical analysis revealed a decrease in the density of invasion in the HeLa cell line (De Petrocellis et al., 2011). Cannabinoids are involved in inhibiting all cancers related to the female reproductive system: cervical cancer, ovarian cancer, gynaecological cancer, and endometriosis. This was proved by performing different practical work by using cannabinoids on the HeLa cell line in animal models (Dawson et al., 2017). By inducing oxidative stress, ceramide production upregulates the formation of ROS and enhances ERS production (Calvaruso et al., 2012). Overall, ROS, ERS oxidative stress, and ceramide together cause apoptosis autophagy by inducing pathways resulting in the inhibition of mTOR/pAkt, which leads to apoptosis (Salazar et al., 2009). Apoptosis may be led by the cAMP pathway, which modulates the Ca^2+^ channels, or by the signalling pathway MAPKs or P38MAPK. Caspase can be activated by the association of cannabinoids with receptor activation, by stimulating the mitochondrial pathway, or by the activation of various substrates leading to the morphologic features of apoptosis (Peter and Krammer, 2003). The interaction of cannabinoid with TRPV modulates apoptosis through the mitochondrial pathway by increasing the ROS level and oxidative stress (Maccarrone et al., 2000). Cannabinoids, rather than receptor interaction, interact with lipid rafts of the cell membrane, leading to the P13K/AKT pathway by downregulating the pathway related to apoptosis (Scuderi et al., 2011). A high level of ER stress increases the production level of mediators related to ER stress. Mediators CHOP, P8, and TRB-3 are responsible for apoptosis via the mitochondrial pathway (Pellerito et al., 2010). Reduction in AKT and upregulation of ROS lead to the upregulation of apoptotic proteins (Salazar et al., 2009; Eichele et al., 2009). Some studies reported about the antimigrative characteristics of cannabinoids on the cancer cell line of HeLa by inducing TIMP-1 through the activation of MMP inhibitors for enhancing the anti-invasive effects. The study reveals that in the exposure of the HeLa cell line to cannabinoids, TIMP-1 acts as a mediator, which upregulates the anti-cancer activity of cannabinoids within 12–24 h of inoculation (Ramer et al., 2007). Various findings demonstrate the potent anti-invasion activity of TIMP-1 by inhibiting MMP in the cervical cancer HeLa cell line by cannabinoid THC (Ramer and Hinz, 2008). Cannabinoids are involved in pp42/44, and pp38 upregulation is involved in TIMP-1 upstream regulation. Upregulation of TIMP-1 forms the MMP-9 and MMP-2, which are inhibitory complexes that initiate anti-invasion of cervical cancer (Ramer et al. 2013). In various signalling pathways of cannabinoids, ROS play a crucial role in activating caspase, which stimulates oxidative stress, leading to apoptosis in carcinoma cell lines (Wondrak, 2009). In prostate and cervical cancer cell lines HeLa CBD downregulates the viability of cells, the growth of tumors, the proliferation of cells, metastasis, invasion, and upregulates the apoptosis in carcinoma cells (Lukhele and Motadi, 2016). It was demonstrated that in lung and cervical cancer, antagonist effects of receptors TRPVI, CB1, and CB2 reversed the antitumor effects induced by CBD. According to Appendino et al. (2008) Although the anticancer effects of cannabinoids have been reported for many years, recently, cannabinoids have appeared as a remarkable novel agent for pharmacological mediation to fight against numerous cancers. Cannabinoids act as the most potent targets for inducing autophagy, apoptosis, and reducing proliferation and invasion in the cancer cell line of HeLa (Ramer et al., 2010).

A protein called PD-1 is a component of the immune checkpoint, a route that tumor cells can control to evade immune system destruction, advance, and withstand therapy (Gong et al., 2018). CBD and THC act on numerous cellular signalling pathways, suggesting that these molecules could be used as ways to adjust the immune system to increase the effectiveness of immune checkpoint inhibitors. Current research has focused on the effects of cannabinoids and the tumor microenvironment on the specific downregulation of PD-L1 in cancer cells. According to another earlier study by Blasco-Benito et al. (2018), CBD improved immune cell recognition to limit tumor growth and suppress PD-L1 expression in breast cancer cells (Dobovišek et al., 2024). Reduced PD-L1 has been linked to changes in various oncogenic signalling pathways, including the AKT/mTOR pathway, which is frequently disrupted in breast cancer (Wang et al., 2024). Therefore, cannabinoids may work by strengthening the immune system to mediate antitumor effects, thus bypassing one of the major immunosuppressive pathways. Additionally, a number of animal models suggest combined treatment with marijuana and currently available medications based on PD-1/PD-L1 agents (Seltzer et al., 2020).

Cannabinoids promote the entry of cytotoxic T cells into the tumor microenvironment, a vital factor in efficient immunosuppression against tumors. These results imply that the immunosuppressive potential of cannabinoids could be very synergistic with immune checkpoint inhibitors since tumor cells use PD-1/PD-L1 signalling for their own survival (Bao et al., 2018). However, although the therapeutic potential of cannabinoids is attractive, translating these findings into clinical applications is not without its difficulties. The other issue is the correct dosage and formulation of cannabinoids that would produce the highest number of treatments while having reduced side effects because cannabinoids have two-sided effects on different types of cancer models (Massi et al., 2013). Another important direction in the study of cannabinoids is the determination of possible breast cancer subpopulations most likely to respond positively to cannabinoid treatment, especially for tumors that are high in PD-L1 expression or have shown immunotherapy resistance (Stanowska et al., 2022). Therefore, cannabinoids could be effective adjuvants by overcoming tumor evasion mediated by the PD-1/PD-L1 pathway in breast cancer. Given their ability to modulate immunity, reduce PD-L1 expression and increase the efficacy of immune checkpoint inhibitors, more research in clinical practice is warranted.

Furthermore, future research could explore in detail how cannabinoids influence the immune system and how cannabinoids should be administered for the best results. PD-1/PD-L1 is involved in immune checkpoints, whereby cancer cells, through this pathway, inhibit T-cell functions and enhance cancer cell growth (Keir et al., 2008). The current investigation also established a combinational approach involving CBD, THC and humulene whereby the cancer antigen PD-L1 was suppressed, hence restarting T-cell-mediated immune responses. These results are consistent with recent investigations that have underscored the possibility that the ability of cannabinoids to regulate immune checkpoint networks is a promising strategy for boosting antitumor immunity (Gao et al., 2022; Duan et al., 2016). The 8-OHdG levels also greatly decreased in the treatment groups in our study, indicating a decrease in oxidative stress. Research has also revealed that cannabinoids possess antioxidant properties and that they work against cancer through their ability to prevent oxidative stress in cancer cells (Nedzamba, 2022). As 8-OHdG levels are also decreased by combination therapy, tumorigenesis and DNA repair capabilities might improve”. Tumor necrosis factor-alpha is an inflammatory cytokine that has been established to actively induce inflammation in cancer, thereby promoting tumor formation (Balkwill, 2006).

The present study also revealed that TNF-α levels are reduced with combination therapy. These results are similar to those of other works that describe the interaction of cannabinoids and specific terpenes, including humulene, which has been shown to potentially regulate TNF-α signalling (Monyela et al., 2024). Regulated-on-activation normal T-cell expressed and secreted (RANTES) has a role in modulating immunity, mostly through inflammatory processes such as chemotaxis and the suppression of the growth of cancer cells (Mafi et al., 2022). We found that combination therapy lowered MMP-9, which may prevent cancer cell invasion in breast cancer. Similar observations have been reported where cannabinoids suppress MMP-9 activity, which in turn decreases metastatic properties in numerous cancer types (Blázquez et al., 2008).

Another pro-inflammatory cytokine, interleukin-6 (IL-6), is known to promote tumor development, metastasize, and prevent therapy (Kang et al., 2021). The decrease in IL-6 identified in this study indicates that cannabinoids and humulene can regulate inflammatory processes. There is evidence that cannabinoids can decrease the production of IL-6 and prevent tumor growth (Torabinejad et al., 2023). AOPPs and AGEs are indicators of oxidation and inflammation, which are generally reported to be high in cancer patients (Ou et al., 2017). The decrease in AOPPs and AGEs in the treatment group suggests that the constituents CBD, THC and humulene H may prevent oxidative stress, which plays an important role in slowing tumor growth. A number of previous studies have indicated that cannabinoids are beneficial antioxidants, and the results of the present study are in agreement with these findings (Guerrero-Alba et al., 2019).

Targets for cancer treatment include programmed cell death protein 1 (PD-1) and its ligand PD-L1, which are implicated in immune checkpoint control and tumor immune evasion. To suppress the immune response, especially T cells, which allow for immunological monitoring and tumor removal, cancers stimulate PD-1/PD-L1 (Keir et al., 2008). Understanding how this pathway promotes tumor growth has led to strategies to prevent T cells’ PD-1 from attaching to the tumor cell’s PD-L, thereby utilizing the immune system to combat malignancy. The PD-1 receptor of activated T cells interacts with PD-L1, which is elevated in a number of malignancies (Zou et al., 2016). This interaction results in the suppression of T-cell proliferation, cytokine production and cytotoxic activity; hence, the tumor escapes immune surveillance. The interaction of PD-1 with PD-L1 inhibits TCR signals that the immune system should use to identify and kill cancer cells (Topalian et al., 2015).

This mechanism has been noted in lung, melanoma and breast cancer, which are characterized by high PD-L1 expression and generally yield a poor prognosis (Muenst et al., 2016). While nivolumab and pembrolizumab, which target the PD-1/PD-L1 pathway, are effective in patients with various cancers, tumor escape continues to be a key concern. Of these, principal resistance might be caused by tumor-intrinsic mechanisms, such as genetic alterations in the interferon-γ (IFN-γ) signalling pathway, on which PD-L1 expression depends (Gao et al., 2016). However, the latter type of immune checkpoint inhibition resistance, known as acquired resistance, also occurs in cancers. This commonly involves increases in other immune checkpoints, such as LAG-3 or TIM-3, in addition to the addition of immunosuppressive cells, such as Tregs or MDSCs (Chen and Mellman, 2017). Preclinical and clinical studies have been performed to overcome the limitations of single anti-PD-1/PD-L1 treatment because of the multifaceted immune resistance mechanisms involved. New findings have revealed interactions between combining PD-1/PD-L1 inhibitors with other immune checkpoint inhibitors, angiogenesis inhibitors or chemotherapies, encouraging the immune response and hindering the escape of tumor cells (Antonia et al., 2018). For example, the concomitant use of ICIs with VEGF inhibitors is effective in treating renal cell carcinoma and hepatocellular carcinoma (Oura et al., 2023). These therapies envisage enhancing T-cell uptake in tumors to enhance the body’s antitumor response. The use of PD-L1 cancer cell expression as a biomarker for PD-1/PD-L1 inhibitor response has attracted attention; however, its accuracy in this context, if used alone, is questionable (Patel and Kurzrock, 2015). Other biomarkers related to a greater mutational number and MSIs that are earmarked by IFN-γ gene expression profiles suggest better outcomes with immunotherapy (Cristescu et al., 2018). The discovery of these biomarkers has been made possible by new technologies in genomics and proteomics, which have enhanced immunotherapy and have become more personalized. To this end, new approaches are being sought to overcome this hurdle, and one such opportunity involves targeting the tumor microenvironment (TME). Tumor-associated macrophages and other noncancerous cells, such as cancer-associated fibroblasts, actively inhibit T-cell functions and enhance the progression of cancer.

They believe that they may be able to do so by altering the environment in which the tumor exists, known as the tumor microenvironment (TME), and the present therapies that target those stromal cells in conjunction with PD-1/PD-L1 could increase the survival of the patient (Mariathasan et al., 2018). Another practical strategy is the use of engineered T cells, such as CAR-T cells, which can be programmed such that they are not affected by PD-1/PD-L1. These modified T cells can remain capable of functional cytotoxicity at immune checkpoints with high PD-L1 levels, suggesting the issue of immune resistance (Dias et al., 2024).

## 5 Conclusion

Cancer still has no known treatment, and research is being conducted to create lead compounds and precursors that could be used as anticancer medications for 1 day. The goal of this study was to identify natural compounds with anticancer properties. The MTT assay showed that cannabinoids retain anti-proliferative, anti-invasion, and apoptotic effects. IC_50_ upregulates 51%–77.6% of carcinoma cell death. The synergistic effects of cannabidiol, tetrahydrocannabinol, and humulene significantly suppressed PD-1/PD-L1 expression and oxidative stress, suggesting a possible approach for targeting breast cancer resistance. The greatest effect was obtained when all three compounds were combined, suggesting that the immunosuppressive and oxidative stress-modulatory effects of the compounds occurred synergistically. Herein, we report comprehensive findings that may be helpful in designing new combinatory therapeutic strategies for breast cancer via the PD-1/PD-L1 pathway and oxidative stress markers. Further studies and trials are needed to identify more cannabinoid-based treatments and to combine pharmacological and cannabinoid drugs to gain remarkable effects against various cancer treatments.

## Data Availability

The original contributions presented in the study are included in the article/Supplementary Material, further inquiries can be directed to the corresponding author.
