# Supplementary material for: Extraction, GC-MS analysis, cytotoxic, anti-inflammatory and anticancer potential of Cannabis sativa female flower; in vitro, in vivo and in silico
Source: Front Pharmacol. 2025 Feb 11;16:1546062. doi: 10.3389/fphar.2025.1546062 (PMC11850312; doi:10.3389/fphar.2025.1546062)
Supplement: Supplementary file 1 [file Table1.docx]

| **GROUPS** |  | **MEAN±SD (n=10)** | | | | | | |
| --- | --- | --- | --- | --- | --- | --- | --- | --- |
|  | **PD-1**  **pg/ml** | **PDL-1**  **pg/ml** | **8-OHdG**  **pg/ml** | **TNF-α**  **pg/ml** | **MMP-9**  **pg/ml** | **IL-6**  **pg/ml** | **AOPPs**  **(IU/mL)** | **AGEs**  **(AU)** |
| **A** | 50.89±3.84 | 0.565±0.084 | 5.98±1.97 | 19.98±3.87 | 41.85±4.56 | 10.98±1.99 | 0.96±0.54 | 0.95±0.44 |
| **B** | 102.60±10.24 | 2.86±0.99 | 21.45±3.84 | 41.25±4.56 | 61.55±3.40 | 32.54±2.56 | 1.64±0.86 | 1.68±0.61 |
| **C** | 99.65±11.29 | 1.78±0.77 | 28.65±2.88 | 34.65±1.99 | 70.50±2.98 | 19.87±1.44 | 2.56±0.29 | 1.76±0.59 |
| **D** | 45.99±4.57 | 2.56±0.59 | 18.78±4.78 | 31.44±3.49 | 51.59±5.50 | 32.56±2.80 | 1.84±0.84 | 1.56±0.49 |
| **E** | 91.33±8.50 | 1.44±0.77 | 23.87±7.78 | 34.47±2.97 | 60.56±7.41 | 18.95±1.89 | 1.67±0.54 | 1.76±0.95 |
| **F** | 98.64±7.22 | 1.88±0.84 | 17.70±2.96 | 28.89±6.17 | 45.58±3.94 | 14.85±3.87 | 2.44±0.46 | 1.81±0.66 |
| **G** | 80.67±14.33 | 1.99±0.92 | 15.61±2.89 | 26.77±4.59 | 45.69±7.57 | 41.85±1.77 | 2.36±0.56 | 1.44±0.84 |
| **H** | 61.45±10.22 | 2.63±0.67 | 14.55±4.87 | 31.79±3.79 | 47.96±6.54 | 31.36±3.66 | 1.86±0.82 | 1.89±0.87 |
| **I** | 54.49±8.78 | 0.651±0.095 | 13.94±1.99 | 20.80±4.64 | 14.99±5.69 | 14.61±4.56 | 1.83±0.64 | 0.97±0.39 |
| **J** | 88.29±14.39 | 0.955±0.099 | 12.44±5.00 | 25.62±3.67 | 48.68±7.21 | 61.55±1.99 | 1.93±0.63 | 1.77±0.98 |
| **LSD (0.05)** | 4.56 | 1.15 | 4.49 | 4.47 | 7.89 | 6.67 | 0.79 | 0.95 |
| **p-VALUE** | 0.064 | 0.056 | 0.041 | 0.037 | 0.029 | 0.049 | 0.041 | 0.009 |

**SUPPLEMENTARY TABLES**

**Table S1. Response Of Cannabidiol, Tetrahydrocannabinol, And Humulene Following 7,12- Dimethylbenz (A)Anthracene (Dmba) Induced Breast Cancer In Rat**

- Mean values ± Standard deviation (SD)

- n = 10 rats in each group

- Values not sharing a common letter differ significantly (p<0.05)

- LSD (Least significant difference)

- ns = Non-significant

| GROUPS | **TREATMENT DURATION** | | | **MEAN±SD**  **LSD=21.58** |
| --- | --- | --- | --- | --- |
|  | **7-10 Week** | **11-14 Week** | **15-18 week** |  |
| **A** | 000±000 | 000±000 | 000±000 | 000±000 |
| **B** | 789.75±129.69 | 994.01±137.74 | 1112.73±122.97 | 965.49±137.04 a |
| **C** | 785.20±128.75 | 868.71±233.75 | 939.41±104.89 | 864.44±139.51 a |
| **D** | 774.23±136.78 | 862.14±121.89 | 941.82±73.21 | 859.39±124.48 a |
| **E** | 862.86±242.49 | 953.94±132.97 | 734.45±95.02 | 850.42±232.46 a |
| **F** | 747.64±156.52 | 943.85±144.71 | 1034.35±105.19 | 908.61±175.55 a |
| **G** | 753.98±129.80 | 649.74±155.62 | 630.56±109.08 | 678.09±133.24 ab |
| **H** | 645.55±175.51 | 542.17±143.37 | 530.37±102.56 | 572.69±127.67 b |
| **I** | 540.58±133.04 | 437.75±139.77 | 422.26±104.53 | 466.86±155.13 bc |
| **J** | 521.55±150.21 | 419.44±244.55 | 410.63±105.60 | 450.54±153.29 bc |
| **MEAN±SD**  **LSD=102.09** | 713.48±153.64 a | 961.75±161.59 b | 750.73±98.88a | p-value(<0.017) |

**Table S2. Weight Of Mammary Tissue Were Calculated as Standard Deviations (Mean±SD).**

Values are mean ± SD from 10 rats in each group

Values not sharing a common letter differ significantly at p<0.05
